# Acknowledgement to Reviewers of *Medicines* in 2019

**DOI:** 10.3390/medicines7010006

**Published:** 2020-01-19

**Authors:** Medicines Editorial Office

**Affiliations:** MDPI, St. Alban-Anlage 66, 4052 Basel, Switzerland

The editorial team greatly appreciates the reviewers who have dedicated their considerable time and expertise to the journal’s rigorous editorial process over the past 12 months, regardless of whether the papers are finally published or not. In 2019, a total of 103 papers were published in the journal, with a median time to first decision of 20 days and a median time from submission to publication of 36 days. The editors would like to express their sincere gratitude to the following reviewers for their generous contribution in 2019:
Abdelbasset, Walid KamalLu, DongdongAbdeltawab, NourtanLu, Jeng-WeiAeddula, Narothama ReddyLushington, GerryAi, YongMacauley, Matthew S.Alam, Md BadrulMadonov, PavelAl-Horani, Rami A.Mai, SabineAlsaab, HashemMancianti, FrancescaAltemimi, AMMAR.BManini, Mhd LouaiAncuceanu, Robert ViorelMarchini, CristinaAnsari, ShariqMartínez-Campa, CarlosArgenta, PeterMartins, NataliaArmijos, ChabacoMassari, SerenaAsakawa, TetsuyaMasulli, MariaÅstrand, AlexanderMatys, JacekBarreto, MariaMayor, DavidBarton, Matthew JMcCombe, PamelaBasso, DanielaMcConnell, TraceyBenson, Al B.Melini, ValentinaBeths, ThierryMenezes, Irwin Rose AlencarBiagi, MarcoMenna, MarialuisaBijak, MichałMetwally, MayadaBirlea, MariusMiceli, NataliziaBlanco-Salas, JoséMikula, MarioBogdański, PawełMimaki, YoshihiroBooken, NinaMisery, LaurentBranca, FerdinandoMishra, AbheepsaBrennan, FrankMnif, WissemBreuner, Cora ColletteMo, FeiBrueggen, Marie-CharlotteMogielnicki, AndrzejBrunetti, GiacominaMohr, AndreaBubici, GiovanniMong, Mei-ChinBurns, RyanMonga, VarunCangkrama, MichaelMonteiro, MarianaCapasso, RaffaeleMorais, NunoCarr, Catherine ElizabethMorello, RoyCelakovska, JarmilaMorgan, JillCen, ShanMortara, LorenzoChang, Yuan ShiunMulas, MaurizioChau, Chi-FaiMuñoz, MiguelChen, Kuo-HuNakamura, TomokiChen, Yung-HsiangNanavati, CharviCho, Hea-YoungNarakathu, Binu BabyCho, Tae-joonNduom, EdjahConti, BarbaraNolan, ClodaghContino, MarialessandraOláh, AttilaCruickshank, Margaret EleanorOmidian, KosarCruz-Chamorro, IvanOthman, Eman M.Da Silva, Luis Cláudio NascimentoPalermo, SaraDamiani, GiovanniPaneth, AgataDatta, PoulamiPark, HangueD’auria, EnzaParodi, EmiliaDavelaar, Eddy J.Pastori, DanieleDe Matteis, RitaPeyton, David H.De Vita, AlessandroPfeffer, GeraldDeng, XiaoranPolańczyk, AndrzejDenkova-Kostova, RositsaPoling, MikaelaDesai, Umesh R.Primack, William A.Dhanasekaran, MuraliPsurski, MateuszDiaconeasa, ZoritaRahman, Md ToufiqurDíaz Martínez, MargaritaRamachandran, AnupEndo, MakotoRamos-Jiménez, ArnulfoEsparham, AnnaRanadheera, Chaminda SenakaEsposito, Emilio XavierRanzato, EliaEsworthy, SteveRavuri, SudheerEzhov, MaratRelijc, RajkoFernández-Ochoa, ÁlvaroReybrouck, MarkFerriz-Martínez, Roberto AugustoRidiandries, AnisyahFerroni, PatriziaRincón-Sánchez, Ana RosaFine, James BurkeRishi, GautamFischer-Fodor, EvaRodrigo, LuisFiz, FrancescoRogobete, Alexandru FlorinFleckenstein, JohannesRomano, StefanoFousteris, ManolisRomano-Chernac, Fabian B.Frass, MichaelRose, KlausFrigstad, Svein OskarRostoker, GuyFürnsinn, ClemensRotblat, BarakGallo, MonicaRout, BhimsenGaspar, Maria ManuelaRuiz-Gómez, GloriaGawlik-Dziki, UrszulaS. Gandhi, NehaGershan, LynnSaiano, FilippoGervasoni, JacopoSaintigny, PierreGestal, MonicaSalisbury, JosephGolianu, BrendaSantos, HugoGolubovskaya, VitaSaravanakumar, KandasamyGomes, Luciana CalheirosSavoia, PaolaGonzález-Vallinas, MargaritaSchauss, AlexanderGorman, GregSchellenberg, E. GlennGrieco, DomenicoSchirmer, BastianGuzek, DominikaSchlaeger, Judith M.Halkia, Evgenia A.Scholkmann, FelixHamasaki, HidetakaSeca, SusanaHaponiuk, JózefSerralheiro, Maria LuísaHarding, KatharineShamay, YosefHashiguchi, AkikoShear, NeilHeise, DanielShiao, Chih-ChungHolmes, NeilShimada, YasuhitoHook, IngridSilverman, Michael JHossain, Md KamalŚmigielski, Krzysztof B.Howes, Laurence GuySmith, Leonard B.Huang, Ren-YeongSorokina, I. V.Ikeda, KazuyoshiSpałek, KrzysztofInokuchi, TsutomuSpriano, GiuseppeIsoherranen, KirsiSt Hill, CatherineIsola, GaetanoStauber, Rudolf E.Jacob, FrancisSuen, Jacky Y.Jakobek, LidijaSun, GuohuiJanakiraman, HarinarayananTan, Bee KangJaradat, NidalTanaka, Tim HideakiJordan, ElizabethTatu, Alin LaurenţiuJun, Joon-YoungTatullo, MarcoJurjus, Rosalyn A.Tedesco, IdoloKaliora, Andriana C.Teschke, RolfKandylis, PanagiotisThompson, BillKasarda, RadovanToiu, AncaKatagiri, KazumotoTsagareli, Merab G.Khairullina, VeronikaTseng, Chih-HuaKikuchi, HidehikoTudela, JoseKim, Chuk YoungTurrini, EleonoraKim, Hye OneUllsten, AlexandraKim, Jeong HeeUnchwaniwala, NuruddinKipp, MarkusUrzua, AlejandroKolisek, MartinValentovic, MonicaKong Thoo Lin, PaulVamanu, EmanuelKostecka, MalgorzataVan De Ven, RienekeKubicova, LenkaVan den Noort, MauritsKumar, AmitVargas-Murga, LilianaKumar, NitinVaughan, Christopher W.Kumar, PraveenVelez, ZéliaKunutsor, Setor K.Veneri, Diana A.La Mendola, DiegoVishe, MaheshLai, SilviaVolicer, LadislavLai, Wing-FuWall Medrano, AbrahamLast Name, First NameWallace, Nicholas A.Lauritano, DorinaWan, ShibiaoLayek, BuddhadevWanchai, AusaneeLazzarato, LorettaWang, XinyuLee, Wei-ChiehWdowiak, KamilLee, Yu-ChenWeighardt, HeikeLeon, FranciscoWhitney, CoryLes, FranciscoXu, WilliamLesyk, Roman B.Yaksh, TonyLewinska, AnnaYan, MaryLi, FuyangYang, Wei-hsiungLidon, FernandoYoussefian, LeilaLifehouse, ChrisYuan, LiyunLiu, ZhiqingYuan, ZhihongLópez, Jesús AdriánZabetakis, IoannisLorenzi, VanninaZucca, PaoloLosurdo, Giuseppe


